# Self-compassion and repetitive thinking in relation to depressive mood and fear of the future: An investigation during the 2020 coronavirus pandemic in semiprofessional football players

**DOI:** 10.1007/s12662-021-00712-y

**Published:** 2021-03-04

**Authors:** Petra Jansen

**Affiliations:** Faculty of Human Science, University of Regensburg, Universitätsstr. 31, 93053 Regensburg, Germany

**Keywords:** Rumination, Worry, Anxiety, Soccer, Mindfulness

## Abstract

The coronavirus pandemic has had a high impact on mental health. Also, semiprofessional football players are strongly affected by the coronavirus disease 2019 (COVID-19) because training during the lockdown phase has been forbidden. It was the primary goal of this study to investigate if those athletes suffer from a depressive mood and fear of the future. Furthermore, the question was asked whether the psychological variables of self-compassion and repetitive thinking are related to this. A total of 55 semiprofessional football players completed a demographic questionnaire with questions related to depressive mood and fear of the future, and a rumination-, worry- and self-compassion scale. The results show an association between the negative scale of self-compassion and depressive mood as well as fear of the future. Whereas depressive mood was predicted by self-compassion, fear of the future was only indirectly predicted by self-compassion by the mediating effects of repetitive thinking. Also, in semiprofessional football, self-compassion interventions might be a useful tool in difficult times.

**Supplementary Information:**

The online version of this article (10.1007/s12662-021-00712-y) contains supplementary material, which is available to authorized users.

## Introduction

The coronavirus pandemic has a great impact on everyone’s life. In general, a lot of (but not all) people react to pandemics with maladaptive behaviors, emotional distress, and defensive responses (Taylor, 2019). Wang et al. (2020) already demonstrated a strong association between physical symptoms and psychological impacts on the coronavirus pandemic (see also Cao et al., 2020). In Germany, during the beginning of the pandemic, organizational sports have been forbidden, a restriction that might affect athletes immensely. Thus, the main focus of this study is to investigate whether psychological trait variables of self-compassion and repetitive thinking are related to negative state variables during the coronavirus pandemic in semiprofessional football players.

## Self-compassion and repetitive thinking in athletes

Self-compassion describes compassion toward oneself, while suffering and can be differentiated into the three positive aspects with three negative counterparts of self-kindness vs. self-judgment, mindfulness vs. overidentification and common humanity vs. isolation (Neff, 2003). There has been evidence that the trait self-compassion consistently predicts psychological well-being (Neff & Germer, 2017). In a German population the negative scale of self-compassion was related with measures of distress (Coroiu, Kwakkenbos, Moran, Thombs, & Albani, 2017).

Rumination and worry are considered as two types of repetitive thinking. While ruminating includes the pondering of possible causes for some failures (Nolen-Hoeksema, Wisco, & Lyubormirsky, 2008), it can deepen a depressed mood (e.g. Watkins, 2008). Whereas rumination is mostly focused on past events, worrying is described as repeated thinking about prospective risks and uncertainties (Watkins, 2008). Rumination and worry share common processes, but they also differ in their past and future orientation (Raes, 2010).

In a study with healthy students, Raes (2010) investigated the two aspects of repetitive thinking (rumination and worry) as mediators of the relationship between the trait variables of self-compassion on the one hand and depression and anxiety on the other. The relationship between self-compassion and anxiety was mediated through the aspect of brooding from the rumination scale and worrying, whereas the mediating effect of worry on anxiety was higher than the rumination aspect.

Regarding the sport context, it has been shown that low self-compassion (as well as higher fears of compassion and higher feelings of inadequacy) predicted psychological distress in athletes of different competitive levels (Walton, Baranoff, Gilbert, & Kirby, 2020). Furthermore, self-compassion was negatively correlated with self-criticism in female athletes (Killham, Mosewich, Mack, Gunnel, & Ferguson, 2018) and positively correlated to eudaimonic well-being in young female athletes (Ferguson, Kowalski, Mack, & Sabiston, 2014). Regarding repetitive thinking, firstly a direct relationship between rumination and action orientation in competitive athletes has been demonstrated (Kröhler & Berti, 2019). Secondly, between 27 and 36% of the variance in ski flying results could be accounted for by the amount of worry (Sklett, Loras, & Sigmundsson, 2018). However, until now the concepts of self-compassion and repetitive thinking have not been related in the sport context.

## Purpose and hypothesis

Raes (2010) provided evidence for a relationship of self-compassion and the mediating effect of repetitive thinking to the trait concepts of anxiety and depression. However, this relationship has not been investigated with regard to the state variables of depressive mood and fear of the future in a challenging situation. This study will be conducted in the sport context because the concepts of self-compassion (e.g. Mosewich, 2020) and repetitive thinking play an important role in this area (e.g. Kröhler & Berti, 2019). Semiprofessional football players were chosen because it has been shown that in this group the anxiety values were higher compared to the normal values in nonathletes of the same age group indicating a vulnerable group for negative psychological symptoms (Jansen, Lehmann, Fellner, Huppertz, & Loose, 2019).

The following hypotheses will be investigated:

According to Coroiu et al. (2017) and Raes (2010), a negative correlation between the negative scale of self-compassion and the state variables of depressive mood and fear of the future as well as a positive correlation between this scale and the two aspects of repetitive thinking is assumed.

In an exploratory manner it is investigated if the state variable of depressive mood can be predicted by self-compassion and repetitive thinking.

According to Raes (2010), it is hypothesized that the relation of self-compassion and fear of the future is mediated by the worry aspect of repetitive thinking.

## Methods

### Participants

Because the effect size of the correlation between self-compassion and depressive mood (anxiety) was *r* = −0.55 (*r* = −0.75) (Raes, 2010), we assume an effect size of *r* = 0.55, an alpha-level of *p* < 0.005 (Bonferroni corrected) and a power of *1* − *β* = 0.95. A power analysis (G‑Power, Faul, Erdfelder, Lang, & Buchner, 2007) for the correlation resulted in a total of *N* = 50 to detect significant effects regarding the correlation between self-compassion, rumination, and worry on the one hand and fear of the future, depressive mood, and loss of interest on the other.

Fifty-six out of 95 semiprofessional football players (league 5–7) from a larger sample completed the questionnaires and 55 were included in the analysis (32 men [mean age = 23.91, range 16–35 years, *SD* = 5.15] and 23 women [mean age = 22.83, range 16–46 years, *SD* = 6.71]; Table 1).

**Table 1:** Demographic data of the participants

	Men	Women	*P*
Age	23.91 (5.15)	22.83 (6.71)	0.502^a^
Years of playing football	17.87 (5.03)	16.00 (6.83)	0.251^a^
Frequency of training (per week)	2.90 (1.24)	2.43 (0.90)	0.132^a^
Number of competitions (per year)	22.21 (18.9)	24.67 (17.97)	0.649^a^
Mindfulness activity (Yes/No)	*21.87%	*8.7%	0.193^b^
Do you like to be alone? (Yes/No)	*40.62%	*30.43%	0.438^b^

For *age, years of playing football, frequency of training per week* and *number of competitions per year*, both with respect to the participation in the semiprofessional leagues, mean and standard deviation were presented

*For *mindfulness activity* and the *loneliness-question* the frequencies for the Yes-answer were given

^a^t‑test

^b^Chi-square

### Material

#### Demographic questionnaire

In the demographic questionnaire, gender, age, how long they practice football in years, the training frequency per week, the number of competitions in one year, the attendance to mindfulness activities, and the preference to be alone were registered (Table 1).

#### Self-Compassion Scale

The Self-Compassion Scale (SCS; German version: Hupfeld & Ruffieux, 2011) comprises the three positive elements of self-kindness, common humanity, and mindfulness, and the three negative aspects of self-judgment, isolation, and over-identification (Neff, 2003). Responses for the 26 items had to be given on a scale from 1 (almost never) and 5 (almost always). Since for the German scale the total score was not justified, the positive and negative scale were separately used according to the recommendation of Coroiu et al. (2017). Cronbach’s alpha in this study for the positive scale (negative scale) was 0.87 (0.90) which is in accordance (positive scale 0.88, negative scale 0.87) with the study of Coroiu et al. (2017).

#### Rumination–Reflection Questionnaire

The Rumination–Reflection Questionnaire (RRQ; German version: König, 2012) has been developed by Trapnell and Campbell (1999) to examine how often the participants ruminate and reflect on their past. Cronbach’s alpha for the rumination scale, with a maximum of 60 points reflecting a high rumination, was 0.90. Cronbach’s alpha for the German rumination scale was 0.68 in this study.

#### Penn State Worry Questionnaire

The Penn State Worry Questionnaire (PSWQ; German version: Glöckner-Rist & Rist, 2014) consists of 16 items measuring worry (Meyer, Miller, Metzger, & Borkovec, 1990). A value between 1 (almost never) and 5 (almost always) is assigned to a response depending upon whether the item is worded positively or negatively. The test comprises 16 items. A maximum of 80 points could be achieved and reflects a high worry. Cronbach’s alpha for the PSWQ was 0.72 in this study.

#### Depressive mood—questions

With two questions, participants were asked if they felt depressed (1 = yes, 2 = no) and were burdened by lack of interest (1 = yes, 2 = no). Those two questions were taken from a primary care evaluation of mental disorders and showed good sensitivity and reasonable specificity for screening for depression (Arroll, Khin, & Kerse, 2003). In this study, the sensitivity and specificity were 80% and 85%, respectively.

#### Fear of the future—questions

The fear of the future was retrieved with the following three questions:

- How anxious do you feel in the coronavirus pandemic situation?
- How worried are you about the future?
- How much do you think corona will influence your future life?

Responses were provided using a Likert scale from 1 (not at all) to 7 (very much). The mean of the three answers was calculated for the total score. Cronbach’s alpha of this short questionnaire was 0.76.

### Procedure

Participants completed the measures during the lockdown period between the 1st and 14th of May 2020. The online study has been implemented at SoSciSurvey (drop-out rate 41.1%). Participants were recruited through a newsletter from the Bavarian football association. They subsequently completed the tests in the order presented: the demographic questionnaire with the depressive mood and fear of future questions, the Self-Compassion Scale (SCS), the Rumination-Reflection Questionnaire (RRQ) and the Penn State Worry Questionnaire (PSWQ)

### Statistical analysis

First, correlations (Pearson and point-biseral) between the positive and negative scale of self-compassion and (a) rumination, (b) worry, (c) depressive mood, (d) loss of interest, and (e) fear of the future were conducted.

Second, logistic regressions were calculated for the “depressive mood” and “loss of interest” questions with self-compassion, rumination, and worry as predictors. Third, a mediation analysis using the Process Analysis of Hayes (2018) was conducted with rumination and worry as predictors. This analysis uses ordinary least squares regression, yielding unstandardized path coefficients for total, direct, and indirect effects. Bootstrapping with 5000 samples together with heteroscedasticity consistent standard errors were employed. The effects were regarded as significant when zero was not included in the confidence interval.

## Results

### Correlations

Correlations are presented in Table 2. Only the negative scale of self-compassion correlated significantly with the depressive mood, loss of interest and fear of the future questions and was considered in the following analyses.

**Table 2:** Correlations between the psychological variables

*–*	Self-Compassion positive	Self-Compassion negative	RRQ	PSWQ	Depressive mood	Loss of interest	Fear of the future
Self-Compassion positive	1	−0.297*	−0.268*	−0.327*	0.247	0.220	−0.025
Self-Compassion negative	–	–	0.569**	0.691**	−0.425**	−0.475**	0.433**
RRQ	–	–	1	0.686**	−0.243	−0.247*	0.188
PSWQ	–	–	–	1	−0.329*	−0.397**	0.528**
Depressive mood	–	–	–	–	1	0.617**	−0.523**
Loss of interest	–	–	–	–	–	1	−0.366**
Fear of the future	–	–	–	–	–	–	1

*RRQ* Rumination–Reflection Questionnaire, *PSWQ* Penn State Worry Questionnaire

*significant, *p* < 0.05; **significant, *p* < 0.01

### Logistic regression

Two logistic regressions were performed to ascertain the effects of the negative scale of self-compassion, rumination, and worry on the likelihood that participants show (a) a depressive mode, and (b) a loss of interest. The logistic regression model for the depressive mood was statistically significant *χ*^*2*^ (3) = 10.89, *p* = 0.012. The model explained 25% (Nagelkerke R2) of the variance in depressive mood and correctly classified 72.7% of cases. Only self-compassion (*p* = 0.045) but not rumination (*p* = 0.950) and worry (*p* = 0.830) reached significance. Also, the logistic regression model for the loss of interest was statistically significant *χ*^*2*^ (3) = 14.20, *p* = 0.003. The model explained 33.0% (Nagelkerke R2) of the variance in loss of interest and correctly classified 78.2% of cases. Again, only self-compassion (*p* = 0.037), but not rumination (*p* = 0.967) and worry (*p* = 0.629) reached significance.

### Mediation model

A mediation analysis was performed to analyze whether self-compassion predicts fear of the future and whether the perceived rumination and worry would mediate the direct path. An effect of self-compassion on the fear of the future was not observed *β* = 0.354, *p* = 0.115, but on rumination *β* = 6.82, *p* < 0.001, and worry, *β* = 11.19, *p* < 0.001. Self-compassion has only an indirect effect of the fear of the future due to the mediation effects of worry *ab* = 0.794, 95% confidence interval (CI) [0.325, 1.359], and rumination *ab* = −0.3719, 95% CI [−0.751, −0.047].

## Discussion

The results carved out that the depressive mood and loss of interest was predicted by the negative scale of self-compassion. According to the hypothesis, the relation of self-compassion and the fear of the future was mediated by the worry as well as the rumination aspect of repetitive thinking, which is in line with the study of Raes (2010).

### Self-compassion as a psychological tool in an uncertain time for semiprofessional athletes

The conclusion that the negative scale of self-compassion predicts depressive mood and loss of interest fits well with the result that high (total) self-compassion was related to a lower depression rate (Bakker, Cox, Hubley, & Owens, 2019). Thus, it might be worth to consider the reduction of the negative scales of self-compassion as a relevant intervention during other critical times, like injuries (Jansen et al., 2019) or change of clubs or the status for semiprofessional athletes. This can be trained for example by the realization and changing of negative beliefs (Neff & Germer, 2019). However, our results also showed that repetitive thinking and self-compassion are related and that the relation between the negative scale of self-compassion and the fear of the future was indirectly mediated by repetitive thinking. This result is in line with the study of Raes (2010) in healthy students. According to the study of Coroiu et al. (2017), the use of the positive and negative scale of self-compassion provides different results in the German sample. Further studies should be conducted which investigate the relationship of both scales to positive psychological states as for example positive affect.

### Limitations and conclusion

Beside the above mentioned strength of the study limiting factors exist. The first is the selection of the participants. Only those who completed the questionnaires could be included in the study, which caused a selection bias and hindered the examination of possible existing gender differences (Amemiya & Sakairi, 2020). Furthermore, depressive mood and loss of interest were only investigated with the help of a validated screening instrument; the reliability of the German rumination scale is questionable. Despite the correlational nature of the study (we could not be sure that the depressive mood and the fear of the future are due to the coronavirus pandemic and is independent of the family and work situation as well as the time of data acquisition during pandemic), causal conclusions could not be drawn.

However, the results give a hint that practical strategies resulting from the concept of self-compassion, as calming one-self down or journaling emotion can be applied in the sport context^1^ (Frentz, McHugh, & Mosewich, 2020). Furthermore, it would be interesting to investigate the relationship of psychological trait variables as self-compassion and repetitive thinking and psychological state variables in professional football players who experience greater pressure on the one hand but have received more assistance from the government on the other hand, for example through the permission to begin training using hygiene concepts.

## Supplementary Information

Practical applications of self-compassion

## Footnotes

1 For practical application see supplementary material.
