# Supplementary material for: Self-compassion and repetitive thinking in relation to depressive mood and fear of the future: An investigation during the 2020 coronavirus pandemic in semiprofessional football players
Source: Ger J Exerc Sport Res. 2021 Mar 4;51(2):232–6. doi: 10.1007/s12662-021-00712-y (PMC7930895; doi:10.1007/s12662-021-00712-y)
Supplement: Supplementary file 1 — Practical applications of self-compassion [file 12662_2021_712_MOESM1_ESM.docx]

**Practical side of self-compassion**

There are several ways to improve self-compassion which are presented nicely for the German audience in the book of *Kristin Neff and Christopher Germer (2019). Selbstmitgefühl, das Übungsbuch. Freiburg Arbor*. For the English audience, please see <https://self-compassion.org/category/exercises/#exercises>

Useful practices are loving-kindness meditation, identifying what we really want, practicing compassion with equanimity, self-compassion break, supportive touch, self-compassion journal, changing critical self-talk.

Self-compassion interventions used in the sport context:

Mosewich, A. D., Crocker, P. R. E., Kowalski, K.C., & DeLongis, A. (2013). Applying self-compassion in sport: An Intervention with women athletes. Journal of Sport & Exercise Psychology, 35, 514-524. Doi: 10.1123/jsep.35.5.514

In this study, the self-compassion intervention group included a psychoeducation session and a series of writing modules to be done over the course of a 7-day period. It is mentioned that self-compassion is an adaptive way to deal with challenges and that it does not mean to get passive. After the introduction athletes were given an example for a writing intervention. They should think about a negative event in sport which appeared over the last week and provide a detailed description of the event. Then they should a) list ways in which other people experience similar events (common humanity), b) write a paragraph expressing understanding, kindness and concern to themselves (self-kindness) and c) describe the event in an unemotional manner (mindfulness).

In the study of Frentz et al. (2020) practical advices were given:

Frentz, D. M., McHugh, T-L., Mosewich, A. D. (2020). Athletes‘ experience of shifting from self-critical to self-compassionate approaches within high-performance sport. Journal of Applied Sport Psychology, 32, 565-684. Doi: 10.1080/10413200.2019.1608332

Practical strategies for athletes were the following: calming themselves down, generating ideas to be solutions-oriented when experiencing a setback, journaling or logging. Athletes mentioned multiple key factors that influenced them of integrating self-compassion, on an intrapersonal level (mental skills), environmental level (e.g. supportive team environments) and organizational level (access to sport psychologists).
